# From Tumor to Network: Functional Connectome Heterogeneity and Alterations in Brain Tumors—A Multimodal Neuroimaging Narrative Review

**DOI:** 10.3390/cancers17132174

**Published:** 2025-06-27

**Authors:** Pablo S. Martínez Lozada, Johanna Pozo Neira, Jose E. Leon-Rojas

**Affiliations:** 1 NeurALL Research Group, Quito 170157, Ecuador; martinez.sebastian2503@gmail.com; 2 PsyBRAIN Research Group, Instituto de Neurociencias, Universidad Católica de Cuenca, Cuenca 10105, Ecuador; johanna.pozon@ucacue.edu.ec; 3 Escuela de Psicología Clínica, Unidad Académica de Salud y Bienestar, Universidad Católica de Cuenca, Cuenca 10105, Ecuador; 4 Cerebro, Emoción y Conducta (CEC) Research Group, Escuela de Medicina, Universidad de las Américas (UDLA), Quito 170125, Ecuador

**Keywords:** brain tumors, functional connectivity, connectome, resting-state fMRI, brain networks, neurosurgery, glioma, meningioma, metastasis

## Abstract

Intracranial tumors such as gliomas, meningiomas, and brain metastases induce complex alterations in brain function beyond their focal presence. Modern connectomic and neuroimaging approaches, including resting-state functional MRI (rs-fMRI) and diffusion MRI, have revealed that these tumors disrupt and reorganize large-scale brain networks in heterogeneous ways. In adult patients, diffuse gliomas infiltrate neural circuits, causing both local disconnections and widespread functional changes that often extend into structurally intact regions. Meningiomas and metastases, though typically well-circumscribed, can perturb networks via mass effect, edema, and diaschisis, sometimes provoking global “dysconnectivity” related to cognitive deficits. Therefore, this review synthesizes interdisciplinary evidence from neuroscience, oncology, and neuroimaging on how intracranial tumors disrupt functional brain connectivity pre- and post-surgery. We discuss how functional heterogeneity (i.e., differences in network involvement due to tumor type, location, and histo-molecular profile) manifests in connectomic analyses, from altered default mode and salience network activity to changes in structural–functional coupling. The clinical relevance of these network effects is examined, highlighting implications for pre-surgical planning, prognostication of neurocognitive outcomes, and post-operative recovery. Gliomas demonstrate remarkable functional plasticity, with network remodeling that may correlate with tumor genotype (e.g., IDH mutation), while meningioma-related edema and metastasis location modulate the extent of network disturbance. Finally, we explore future directions, including imaging-guided therapies and “network-aware” neurosurgical strategies that aim to preserve and restore brain connectivity. Understanding functional heterogeneity in brain tumors through a connectomic lens not only provides insights into the neuroscience of cancer but also informs more effective, personalized approaches to neuro-oncologic care.

## Simple Summary

Intracranial tumors interrupt brain function beyond their focal presence by altering large-scale neural networks. Recent neuroimaging, including resting-state fMRI and diffusion MRI, reveals tumor-induced changes in both structural and functional connectivity. Diffuse gliomas infiltrate networks, while meningiomas and metastases cause disruption via mass effect and edema. These changes often lead to cognitive deficits that reflect global dysconnectivity rather than local damage. Functional heterogeneity depends on tumor type, location, and molecular features such as IDH mutation. Recognizing these disruptions through a connectomic lens enhances neurosurgical planning, prognostic accuracy, and rehabilitative strategies. This review underscores how network-based insights can lead to more personalized and effective neuro-oncologic care.

## 1. Introduction

Mounting evidence indicates that intracranial tumors produce far-reaching disturbances in brain function by disrupting large-scale neural networks [1,2,3]. Unlike a traditional localizationist view, in which a tumor’s effects are confined to an adjacent cortex, a network perspective reveals that even regions distant from the lesion can be functionally affected via disrupted connections [1]. Cognitive deficits in brain tumor patients often reflect global network dysfunction rather than only focal damage [2]. Pioneering clinical observations and electroencephalography (EEG) studies first noted that tumors cause diaschisis, a depression of activity in areas remote from the lesion [3]. Today, resting-state functional MRI and diffusion tractography enable us to map these disruptions systematically as well as changes in the brain’s connectome—the comprehensive map of neural connections [4].

Connectomics is transforming our understanding of brain tumors as diseases of networks rather than isolated masses. Using resting-state functional MRI (rs-fMRI), researchers can quantify functional connectivity (FC), which represents the temporal correlations of activity between regions, and visualize intrinsic resting-state networks (RSNs), like the default mode network (DMN), in patients with tumors [5]. Diffusion MRI provides complementary insights into structural connectivity (SC) by mapping white matter tract integrity [6]. Together, these techniques allow reconstruction of patient-specific brain networks and graph-theoretical analysis of network topology (e.g., small-world architecture and hub connectivity) [7].

Early connectomic studies have revealed that many hallmark properties of brain networks (such as small-world organization) persist even in the presence of large tumors, reflecting the brain’s network robustness [8]. However, tumors do induce characteristic connectivity changes such as reductions in long-range and local connections emanating from the lesion, with distinct patterns depending on tumor location [8]. Critically, certain high-degree hub regions show disproportionate vulnerability, meaning that, when a tumor impacts a network hub, the disturbance to overall network function is much greater [9]. These insights underline why the clinical impact of a tumor often cannot be predicted by lesion size or location alone; instead, it depends on which networks and hubs the tumor has affected locally or afar.

In this review, we examine functional heterogeneity in intracranial tumors from a connectomic and neuroimaging standpoint; we define functional heterogeneity as the variability in the spatial, temporal, and network-level functional consequences of brain tumors, as measured through neuroimaging and connectomic analysis, reflecting differences in tumor type, location, invasiveness, molecular profile, and the brain’s capacity for plastic reorganization. We focus on adult patients and discuss both pre- and post-surgical contexts; pediatric patients were excluded given their distinct neurodevelopmental, molecular, and plasticity-related characteristics of brain tumors, which merit separate treatment in the literature. Gliomas, meningiomas, and brain metastases (three common tumor types with distinct biology) are used to illustrate how different pathologies disrupt neural networks. We integrate evidence across disciplines, including neuroscience (e.g., mechanisms of plasticity and cortical reorganization), neuro-oncology (tumor invasiveness and molecular drivers), and advanced neuroimaging (rs-fMRI connectivity mapping, diffusion MRI tractography, EEG/magnetoencephalography (MEG) functional networks). In doing so, we highlight how tumors can drive network reorganization (for better or worse) and how these changes relate to histomolecular tumor features. We also address the clinical implications of network disruptions for neurosurgical planning, cognitive outcomes, and rehabilitation, before evaluating emerging future directions such as imaging-guided therapies and network-aware interventions. By synthesizing this literature, we aim to provide a comprehensive connectomic perspective on the functional heterogeneity of brain tumors.

We chose to perform a narrative review because we aimed to synthesize recent and influential findings at the intersection of brain tumors, functional connectomics, and advanced neuroimaging. We conducted a targeted literature search using PubMed, Scopus, and Web of Science between January 2000 and March 2025. Search terms included combinations of: “brain tumor,” “glioma,” “meningioma,” “metastasis,” “functional connectivity,” “resting-state fMRI,” “diffusion MRI,” “connectome,” “network plasticity,” and “neuroimaging.” We prioritized studies that provided original neuroimaging data on network-level changes in tumor patients or those offering conceptual frameworks for interpreting such changes. Inclusion was limited to peer-reviewed studies in English and Spanish, with preference for high-impact journals and recent work. We also included landmark earlier studies for historical context or methodological relevance. This narrative review approach was chosen to allow for flexibility in integrating emerging, interdisciplinary evidence across neuroscience, neuro-oncology, and neuroimaging, particularly where structured data are not yet mature enough to support systematic review or meta-analysis methodology.

## 2. Tumor-Specific Patterns of Functional Network Disruption and Reorganization

**Figure 1:**
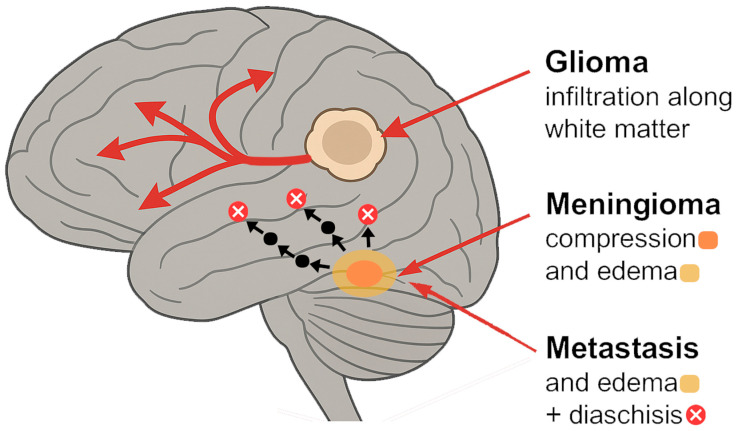
Mechanisms of network disruption by tumor type [10,11,12,13,14,15,16,17,18,19,20,21,22,23,24,25,26,27,28,29,30,31,32,33].

There are multiple reported mechanisms for tumor-specific functional network disruptions. Figure 1 provides a graphical summary of the most important ones. Table 1 provides a structured synthesis of the key mechanisms, patterns of reorganization, histomolecular influences, and clinical implications discussed across Section 2, Section 3, Section 4 and Section 5, organized by tumor type and thematic focus.

### 2.1. Functional Network Disruption in Gliomas

Diffuse gliomas (including infiltrative astrocytomas, oligodendrogliomas, and glioblastomas) exemplify how a tumor can behave as a network disease. Gliomas infiltrate surrounding brain tissue along white matter pathways, as malignant cells migrate preferentially along fiber tracts [10]. This results in widespread alterations of functional connectivity that extend beyond the tumor’s anatomic margins [10]. Resting-state fMRI studies consistently show that glioma patients have abnormal connectivity both proximal and distal to the tumor. For instance, whole-brain rs-fMRI mapping in glioma patients has revealed clear patterns of local and global disconnection that correlate with the functional network distance from the tumor, rather than just spatial distance [11]. In other words, regions strongly connected to the tumor site (functionally) tend to show the greatest connectivity loss, even if they are not immediately adjacent anatomically. Additionally, functional abnormalities (such as reduced within-network coherence or aberrant activity) have been observed in structurally intact brain areas far from the lesion [11]. These remote effects help explain why patients may experience cognitive or neurological deficits that cannot be explained by the tumor’s local effects and invasion alone.

Multiple modalities confirm the global network impact of gliomas. A recent large-scale tractography study demonstrated that gliomas exert histotype-specific alterations on the Inferior Fronto-Occipital Fascicle (IFOF), with glioblastomas and low-grade gliomas causing significant reductions in fascicle volume, length, and fractional anisotropy, consistent with both infiltrative and destructive patterns of network disruption. In contrast, brain metastases primarily caused displacement due to peritumoral edoema, while meningiomas showed no significant effect on IFOF integrity, underscoring the differential impact of gliomas on structural connectivity [12]. Furthermore, a recent prospective study demonstrated that diffusion-based metrics within the brain adjacent tumor area, specifically fractional anisotropy (FA), mean diffusivity (MD), and tract irregularity (TI), correlate strongly with glioma grade, proliferative index (Ki-67), and extent of resection, offering quantitative insight into peritumoral white matter disruption [13]. Notably, TI emerged as an independent biomarker of tumor aggressiveness, reinforcing the utility of advanced DTI-FT analyses in preoperative connectomic assessment and safe surgical planning [13]. MEG studies have found that gliomas affect oscillatory brain networks across a range of frequency bands. In one analysis, glioma patients were reported to have a globally disturbed network organization at rest, with changes in network metrics like clustering coefficient and path length related to their cognitive status [14]. Higher cognitive performance has been correlated with stronger theta-band EEG connectivity in left frontal-parietal regions and an increased clustering and small worldness in theta, delta, and gamma bands (*p* < 0.01); such changes could explain up to 42% of cognitive score variance (adjusted R^2^ = 0.42), suggesting that tumor-induced functional reorganization in these frequencies was maladaptive [12]. Likewise, graph-theoretical analyses of fMRI networks have demonstrated reduced network efficiency and a shift toward a more random network topology in glioma patients [8]. This indicates a loss of the optimal small-world balance between local specialization and global integration. In frontal lobe low-grade glioma patients, this disturbed topology, characterized by reduced clustering coefficient (Cnet) and increased path length (Lnet), was significantly correlated with cognitive impairment, as measured by Montreal Cognitive Assessment (MoCA) scores (Cnet: r = 0.589, *p* = 0.008; Lnet: r = −0.532, *p* = 0.019) [8]. In high-grade gliomas, disruption of interhemispheric connectivity is particularly pronounced, reflecting how a unilateral tumor can perturb bilateral network synchronization. For instance, Mallela et al. analyzed data from rs-fMRI in 24 glioma patients and demonstrated that both global brain activation and interhemispheric connectivity were significantly reduced compared to controls (*p* < 0.05) [15].

One striking aspect of gliomas is the brain’s attempt to compensate or reorganize. Despite significant structural damage (i.e., axonal degeneration in the tumor’s infiltrative path), some functional connectivity may be preserved or rerouted. A recent study found that functional–structural coupling becomes decoupled (dissociated) in glioma patients, while healthy brains normally show strong correspondence between structural connections and functional connectivity [16]. Notably, isocitrate dehydrogenase (IDH)-mutant lower-grade gliomas appear to disturb networks differently than IDH-wildtype tumors like glioblastoma. Connectome analyses revealed that IDH-wildtype gliomas exhibit greater structural connectome aberrations, including widespread reductions in fractional anisotropy (a diffusion measure of white matter integrity) on the same side as the tumor (*p* = 0.002) and contralesionally (*p* = 0.008) [17]. In contrast, IDH-mutant gliomas showed comparatively milder structural disruption and even signs of increased fractional anisotropy (*p* = 0.011) contralesionally (which might reflect compensatory fiber bundle strengthening or less destructive infiltration) [17]. Both types, however, showed loss of normal structural–functional connectomic coherence [17]. These differences align with clinical behavior; IDH-wildtype (usually glioblastomas) are more aggressive and diffusely invasive, consistent with more severe network disintegration, whereas IDH-mutant gliomas (often slower-growing) allow the brain more time to adapt and reorganize its networks [18].

Functional connectivity patterns also correlate with molecular and histological features in gliomas. In an rs-fMRI study of 34 newly diagnosed gliomas (WHO grade II–IV), patients with more aggressive tumors (higher grade or IDH-wildtype) exhibited greater impairment of whole-brain connectivity (Spearman’s ρ = 0.62, *p* < 0.001) than those with indolent tumors [19]. This led to the concept of an “abnormality index”, a quantitative measure of how much a tumor perturbs global functional networks, which tends to be higher in biologically aggressive gliomas [19]. Further subgroup analysis by IDH-1 mutation status showed that IDH-1-wildtype gliomas had significantly higher abnormality index values than IDH-1-mutant tumors (t = 3.17, *p* = 0.003), independent of tumor volume (ANCOVA, *p* = 0.005) [19]. These findings indicate that molecular aggressiveness, not just size, drives connectome disruption. Similarly, connectivity between the tumor-infiltrated region and the rest of the brain has been shown to be inversely related to tumor grade [19]. In one study, oligodendrogliomas (IDH-mutant and lower-grade) had the highest functional connectivity between the tumor and various resting-state networks, whereas glioblastomas (IDH-wildtype, grade IV) had the lowest [20]. This suggests that slower-growing tumors may better integrate into or preserve existing networks, whereas fast-growing glioblastomas cause more complete network breakdown. Paradoxically, a certain degree of functional coupling between the tumor and brain might indicate that some neural pathways remain intact; certainly, recurrent gliomas have been found to maintain significant connectivity to major RSNs even at advanced stages [11].

Intriguingly, functional heterogeneity exists within gliomas as well. High-resolution rs-fMRI has demonstrated that functionally connected subregions can persist inside the tumor mass, especially in glioblastomas [21,22]. These “islands” of intra-tumoral connectivity (i.e., BOLD-signal appearing in areas of a glioblastoma that align with known networks) are invisible in structural MRI but suggest the existence of pockets of relatively intact neurovascular and synaptic activity within even high-grade tumors. The extent of intra-tumoral functional connectivity may have prognostic significance; patients whose glioblastomas showed higher internal connectivity (i.e., more functional voxels within the tumor that were correlated and network-active) could have longer overall survival than those with functionally “cold” tumors [21,22]. These findings imply that not all regions of a malignant tumor equally abolish brain function, and some tumor-infiltrated tissue may retain partial functionality or be metabolically less aggressive, thereby participating in residual networks [21,22]. Overall, gliomas present a spectrum of functional disruption: from extensive network disconnection in the case of infiltrative, high-grade lesions, to partial preservation and reorganization of networks around slower-growing tumors. This heterogeneity underscores the need for individualized mapping of functional networks in each glioma patient.

### 2.2. Functional Network Disruption in Meningiomas

Meningiomas are typically benign, extra-axial tumors arising from the meninges. They often compress the cortex and white matter rather than infiltrating it. Given their generally slow growth and extrinsic location, one might assume that meningiomas cause only local mass effect [23]. However, evidence shows that meningiomas can also induce significant functional network changes in the brain. Many meningioma patients experience subtle but measurable cognitive deficits; for example, deficits in attention, memory, or executive function, even when the tumor is benign and surgically resectable, have been reported [2]. These deficits suggest disturbances in the networks underlying those cognitive domains, potentially caused by the tumor’s presence [2].

A key mediator of network disruption in meningiomas is peritumoral brain edema. Some meningiomas provoke extensive vasogenic edema in the adjacent brain, which can injure glial cells and axons and disturb neuronal signaling [24]. A recent rs-fMRI study introduced the concept of a “dysconnectivity index” for meningiomas, analogous to that in gliomas [24]. Radiological and histological factors such as cortical penetration (RR = 2.067, *p* = 0.0148) and vascular supply from pial-cortical arteries (RR = 2.087, *p* = 0.0082) have been independently associated with increased peritumoral brain edema in meningiomas [24]. In 29 preoperative meningioma patients, whole-brain functional connectivity was quantified and compared to healthy controls. The results showed that meningiomas are associated with global functional connectivity impairments, and importantly, the degree of dysconnectivity correlated strongly with edema volume but not with the actual tumor volume [1]. In patients who had significant edema surrounding the tumor, there was a marked reduction in connectivity across the brain (high dysconnectivity index), whereas tumor size alone did not predict network disruption [1]. This indicates that it is feasible for a small meningioma that causes significant edema to result in more global brain network disturbance than a large meningioma with no edema. Edema likely disrupts networks by elevating intracranial pressure, inducing local hypoxia, and damaging white matter tracts (through increased water content and pressure), thereby disconnecting distant brain regions that normally communicate through the affected area [1].

Clinically, these network-level effects of meningiomas manifest in cognitive testing, with peritumoral edema also correlating negatively with MoCA scores (r = −0.5572, *p* = 0.0025) [1]. This aligns with other reports that meningioma patients’ cognitive deficits often improve after tumor resection, suggesting that the tumor and its associated edema were causative factors [25]. Even World Health Organization (WHO) grade I meningiomas (considered “benign”) can disturb complex networks. MEG studies focusing on postoperative benign meningiomas found that reduced connectivity within the DMN correlated with lower working memory performance in patients [2]. Additionally, graph metrics such as the degree of network hubs (using minimum spanning tree analysis) were altered in meningioma patients and related to cognitive outcomes. These findings highlight that extra-axial tumors can still functionally interact and affect the brain parenchyma, through mechanisms such as cortical compression, altered blood flow, inflammation, and edoema [2].

Interestingly, because meningiomas are usually surgically curable, they offer a model to study network recovery after tumor removal. Preoperative network disturbances in meningioma patients often partially normalize postoperatively as mass effect is relieved [2]. However, in cases where a meningioma significantly involved a network hub or caused prolonged edema-induced damage, some connectivity changes may persist after surgery [1]. For example, if a meningioma compressed the frontal lobe, patients might show decreased connectivity within frontoparietal networks pre-surgery, with gradual restoration of those connections in the weeks to months after resection (paralleling cognitive improvement) [1,2]. In summary, while meningiomas do not infiltrate brain tissue, they can still cause functional disconnection syndromes via secondary effects [2]. The heterogeneity in their network impact is driven largely by edema, tumor location (that determines which networks are affected), and chronicity [1,2,23,24,25]. Recognizing this is important, as even patients with benign meningiomas may benefit from cognitive screening and rehabilitative support due to network perturbations that are not immediately obvious on structural imaging or in broad pre- and post-surgical cognitive assessments.

### 2.3. Functional Network Disruption in Brain Metastases

Brain metastases (secondary tumors from systemic cancer) present another distinct scenario of functional heterogeneity. Metastases are typically well-demarcated intra-axial lesions, often surrounded by edoema [26]. Individually, a small metastasis might seem to affect only a focal area, but it can, nevertheless, perturb brain-wide networks. Patients with brain metastases frequently report diffuse symptoms like “brain fog,” memory difficulties, or slowed processing, even when motor or language functions remain intact [27]. Connectomic studies are beginning to reveal that metastases can alter the global network topology of the brain [27].

One rs-fMRI and diffusion MRI study compared 14 patients with brain metastases (pre-surgical) to healthy controls, analyzing both functional and structural connectivity networks [27]. The patients showed an altered small-world architecture in their brain networks, trending toward a more random network configuration compared to the efficient small-world organization seen in controls. In graph terms, metastases led to decreases in network clustering and increases in path length, indicating disrupted local cliques and less efficient global integration [27]. Moreover, the coupling between functional and structural connectivity was weakened in metastasis patients. In a healthy brain, functional connections are largely supported by underlying anatomical pathways, but in these patients, the usual correspondence was reduced; likely because edema or tumor-induced axonal injury breaks structural links even as some functional correlations persist via polysynaptic or alternative routes [27].

Notably, the impact of metastases on networks is not only subtractive (i.e., breaking down connections) but could also be additive in complexity. The presence of multiple lesions introduces multiple disconnection points, which, in network terms, increases the randomness of information flow [3]. In one study, the authors performed a simulation of the removal of network nodes corresponding to the location of metastases; this simulated “resection” of tumor nodes led to further degradation of network measures, suggesting that the brain had already been compensating to some extent with the tumors in place [27]. Therefore, if the tumors occupy or overlap hub regions, their removal can potentially worsen network efficiency, at least acutely [27]. Indeed, the study found that the degree of network disruption after simulated removal correlated with the number of hub nodes that had been affected by metastases [27]. In practical terms, this means that a metastasis located in a critical hub (i.e., the posterior cingulate cortex of the DMN) could cause major network reconfiguration if that region’s connectivity is lost, whereas a metastasis in a less connected region would have a more localized effect.

From a functional connectivity standpoint, metastases can induce diaschisis-like effects. For example, a metastasis in the right frontal lobe may lead to reduced functional connectivity in contralateral homologous networks or in downstream regions of the motor and executive networks. Resting-state studies have noted that patients with metastases show decreased connectivity within important networks such as the default mode, salience, and executive control networks, proportional to their symptom severity [28]. A retrospective fMRI study of 29 patients with brain metastases versus 29 healthy controls demonstrated significant attenuation of the BOLD signal in the supplementary motor cortex of the metastasis-affected hemisphere during motor tasks [28]. Additionally, metastases often cause bilateral network changes even when unilateral, due to their influence on interhemispheric connectivity. Some research using EEG has observed changes in power and connectivity in regions far from the metastasis, reflecting the brain’s global response to the focal lesion; possibly via loss of input from the lesioned area or widespread neurochemical changes due to tumor-secreted factors [29]. It is also worth considering that the heterogeneity among metastases might be influenced by the type of primary cancer, which may have varying effects on the brain microenvironment. For instance, melanoma metastases often cause hemorrhage and inflammation, which could acutely disrupt networks, whereas slow-growing breast cancer metastases might allow for more gradual reorganization [30,31]. Multiple metastases pose a compounded challenge, potentially fragmenting networks in several locations. However, the brain can show remarkable resilience, rerouting connectivity around lesions [32]. Certainly, clinicians have observed cases where even patients with numerous brain lesions can retain near-normal cognition, presumably thanks to network reorganization and redundancy [33].

Brain metastases disrupt functional networks by introducing new “nodes” (tumor tissue that does not participate in normal connectivity) and/or by damaging connecting fibers and/or connectivity hubs via edema and pressure. This typically yields a more random, less efficient brain network configuration. The extent of disruption depends on factors like lesion location (especially involvement of hub regions or connecting bottlenecks), number of lesions, and associated edema [27,28]. Understanding network changes in metastasis patients is increasingly relevant as improved systemic cancer control leads to more patients living with brain metastases; managing their cognitive function and quality of life requires recognizing that functional network integrity is a key aspect of the disease.

**Figure 2:**
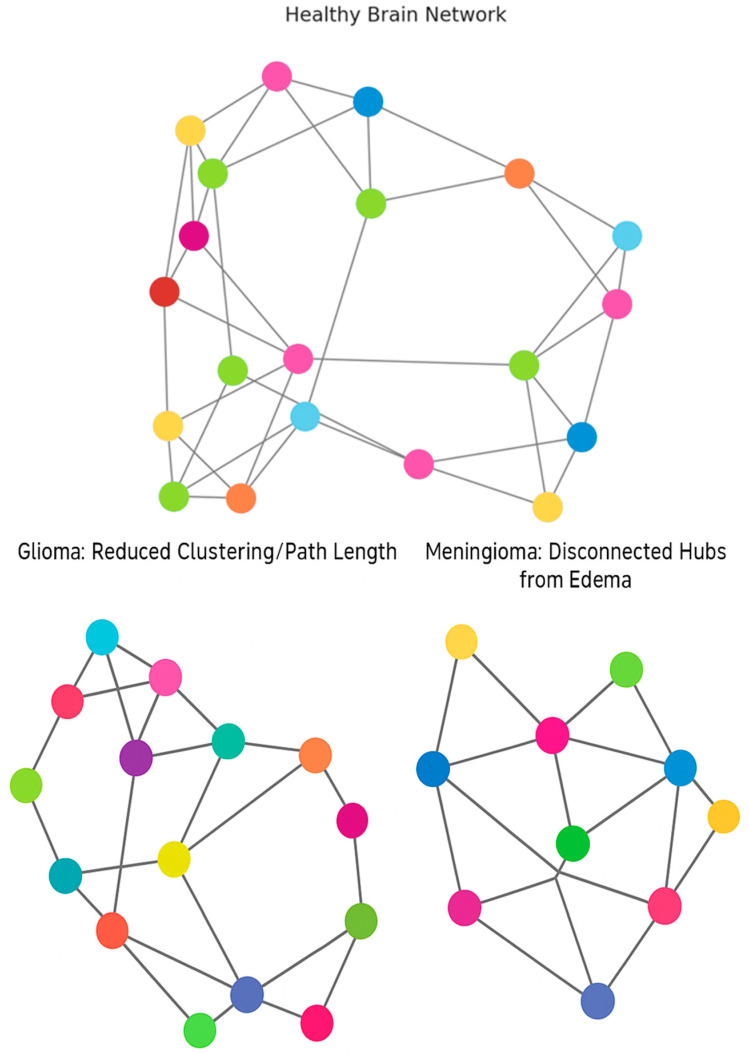
Alterations in Brain Network Topology in Tumor Patients [10,11,12,13,14,15,16,17,18,19,20,21,22,23,24,25,26,27,28,29,30,31,32,33].

Figure 2 provides a comparison of the connectivity profiles that might be seen in brains affected by brain tumors and the related effects on hubs, nodes, and clusters.

## 3. Network Reorganization and Post-Surgical Plasticity

Resection of a brain tumor, while intended to eliminate pathological tissue, is itself a potential perturbation to the brain network. Neurosurgery, therefore, creates an acute change: the sudden removal of tumoral tissue (and sometimes surrounding margin tissue) can restore, disrupt, or redistribute connectivity in complex ways [34]. In these patients, the post-surgical period provides important insights into brain network plasticity and recovery. Studies that perform longitudinal imaging before and after surgery have demonstrated two concurrent phenomena: spontaneous network normalization (as the tumor’s mass effect or infiltrative activity is relieved) and surgery-induced network changes (due to unavoidable cutting of fibers or surgical injury) [35,36]. The balance of these represents an important determinant of the patient’s final functional outcome.

On one hand, successful tumor resection can lead to partial or total recovery of functional networks. For example, a diffusion MRI study of 20 glioma patients noted that subtle structural network differences seen preoperatively in glioma patients (compared to controls) tended to diminish after surgery, implying that the brain’s structural network topology renormalized once the tumor was removed; notably, the degree of preoperative white matter tract involvement significantly predicted postoperative DTI changes (*p* < 0.001), and high-grade tumors were associated with greater rates of subtotal resection (*p* = 0.018) [37]. Similarly, functional connectivity analyses in gliomas have shown that some abnormal connections (or lack of connections) improve post-resection. A longitudinal rs-fMRI study of glioma patients found that several months after surgery, functional connectivity in disrupted networks (such as the default mode network) increased toward normal levels in parallel with the patients’ cognitive recovery [38]. Another study focusing on language-network gliomas observed that as patients recovered language function post-operatively, their resting-state language network connectivity gradually strengthened, essentially reorganizing to support regained function [35]. These improvements likely reflect the brain’s intrinsic plasticity, showcasing the ability of neurons to form new synapses or strengthen alternative pathways when the primary route has been removed or damaged [35].

Importantly, the degree of functional network rebound can vary widely between individuals; factors such as patient age, neuroplastic potential, and the extent of preoperative network damage all play a role [39,40]. However, surgery can also cause new deficits by disrupting networks, especially if the tumor involves critical connections. The concept of “connectomal attack” is useful here: removing a node that is highly central in the network (a hub) will cause a larger drop in network efficiency than removing a peripheral node [39,40]. In practical terms, if a tumor is entangled with a hub (like the thalamus or precuneus), a gross-total resection might require sacrificing some of that hub tissue, thereby markedly affecting network communication [39,40]. Certainly, a study on metastases reported that removal of tumor-infiltrated hubs led to worse network performance in simulated targeted attacks, with attack tolerance (AUC) values significantly lower in patients compared to controls (*p* < 0.04) [41]. Clinically, this correlates with patients who have post-op cognitive or neurological declines despite apparently “successful” resection; the surgery may have unavoidably damaged a network hub or a critical network node [41]. Diffusion tractography can sometimes predict this; for instance, if preoperative tractography shows that the tumor is encasing the arcuate fasciculus (critical for language), even a careful resection might sever this tract and lead to aphasia [42]. In contrast, if the tractography shows displacement but not infiltration, the tract may be preserved, and language networks may quickly normalize after surgery [42]. However, one encouraging finding is that short-term changes in connectivity after surgery often correlate with early neurocognitive outcomes, meaning we can potentially use imaging as a biomarker for recovery [34]. In a 2025 longitudinal study, graph metrics derived from rs-fMRI and DTI were measured before and ~2 weeks after tumor resection; changes reported in these studies were found to be related to neuropsychological test results with a strong correlation (R^2^ ≈ 0.79) between changes in functional network properties and changes in composite cognitive scores [36]. Specifically, increases in functional local and global efficiency were associated with declines in cognitive performance (inversely correlated), whereas increases in network modularity were associated with improved cognition post-operatively [36]. This somewhat counterintuitive result (where higher efficiency correlated with worse cognition) might reflect that an overly “efficient” network shortly after surgery could indicate pathologically reduced segregation (perhaps a sign of network over-simplification after hub loss) [36]. On the other hand, regaining a modular (well-segregated) network architecture could be a marker of healthy recovery of specialized subnetworks. These nuances aside, the key point is that connectivity metrics have the potential to track the trajectory of recovery, likely allowing clinicians to identify patients at risk of poor outcomes and intervene with early rehabilitation [43].

Plasticity after tumor removal can also involve functional reorganization; for instance, after resection in the motor cortex, patients often recruit secondary motor areas in the opposite hemisphere or ipsilateral premotor cortex to regain some functionality [44]. Rs-fMRI might show increased connectivity between these secondary areas and the typical motor network as the patient improves. In connectomic terms, the network finds alternate paths or develops compensatory hubs. Interestingly, one study observed that patients with gliomas showed persistently increased connectivity in the contralesional sensorimotor network (cSMN) compared to controls even postoperatively, suggesting a persistent re-balancing of networks between hemispheres and long-term compensatory reorganization [45]. The clustering coefficient, local efficiency, transitivity, and network vulnerability of the cSMN were significantly higher in the non-deficit group and lower in the deficit group compared to healthy controls (*p* < 0.05) [45]. This could be a form of long-term compensation; essentially, the non-tumor hemisphere increases its internal connectivity to support functions formerly shared with the affected side [45]. It is worth noting that not all postsurgical network changes are beneficial. Some are maladaptive, such as excessive recruitment of frontal networks for a simple memory task (leading to fatigue and inefficiency), or the emergence of hyper-connected “hub” [46]. Tumor surgeries, especially for gliomas, can cause altered functional networks after surgery (like hub overactivation or disinhibition of certain circuits), which can later contribute to seizure genesis [47]. This interplay between networks and excitability is an area of ongoing research, bridging connectomics with electrophysiology.

The post-surgical period in brain tumor patients is often characterized by dynamic network reorganization; functional heterogeneity is evident as some patients’ brains rewire efficiently around the resection cavity, restoring networks and function, whereas others suffer persistent network gaps [35,38]. Understanding each patient’s connectome may help predict these outcomes and tailor interventions (for example, targeted cognitive rehabilitation to strengthen specific network connections, or neuromodulation to encourage plasticity in a weakened network or hub).

## 4. Histomolecular Correlates of Functional Heterogeneity

The structural and functional heterogeneity of brain tumors is partly driven by their underlying histology and molecular genetics. Modern neuro-oncology classifies tumors, like diffuse gliomas, by markers such as IDH mutation, 1p/19q co-deletion, methylguanine-DNA methyltransferase (MGMT) status, and others; features that not only predict prognosis but also influence how a tumor interacts with the brain [48]. Connectomic studies are beginning to link these histomolecular traits to patterns of network disruption.

In gliomas, as discussed, IDH mutation status has emerged as a key determinant of connectomic impact. IDH-wildtype gliomas (which are typically glioblastomas) are fast-growing, necrotic, and diffusely invasive; they tend to cause greater global connectivity loss and more structural network damage [18,49]. IDH-mutant gliomas, often lower grade, show more focal and less severe network disturbances, and sometimes paradoxical increases in certain connectivity measures contralaterally [18,49]. The distinct metabolic environment of IDH-mutant tumors (which produce 2-hydroxyglutarate (2-HG) and often have a less aggressive course) may allow neurons and glia to co-exist longer, preserving function [18,49]. Additionally, oligodendrogliomas (IDH-mutant, 1p/19q co-deleted) often present with chronic seizures, indicating a different kind of network perturbation; hyperexcitability rather than just loss of connectivity [50]. Preoperative seizures occur in 59–74% of IDH-1-mutant patients, significantly more than the 18–34% observed in IDH-1 wild-type cases (*p* < 0.001) [50]. These seizures can drive activity-dependent plastic changes in networks (sometimes hijacking them), and treatments like anti-epileptics or tumor resection can normalize that activity [50]. Functional connectivity is, in fact, found to be higher on average in IDH-mutant lower-grade gliomas than in IDH-wildtype glioblastomas, possibly due to this prolonged interplay with the brain’s baseline activity [50].

Glioblastomas, beyond IDH status, exhibit intra-tumoral molecular heterogeneity (i.e., mesenchymal and proneural regions) that could influence local functional connectivity [51]. For example, a more vascular, infiltrative edge of a glioblastoma might show different rs-fMRI signal characteristics than a hypoxic necrotic core [51]. While connectomics at such fine resolution is challenging, early data like the intra-tumoral connectivity studies suggest that biologically “better” tumor tissue (less hypoxic, more normatively vascularized) correlates with retained BOLD connectivity and better patient survival. In a recent study in 54 gliomas patients, researchers found significant BOLD signal synchronization between the tumor mass and distant brain regions, with tumor-brain functional connectivity strongly correlating with overall survival in both newly diagnosed (r = 0.90–0.96; *p* < 0.001; R^2^ = 81–92%) and recurrent gliomas (r = 0.72; *p* < 0.001; R^2^ = 52%), outperforming standard clinical, radiological, and genetic predictors [51]. Emerging techniques like high-field MRI and PET-MR might further link tumor metabolism or receptor expression with network effects.

In meningiomas, histological grade and secretory properties can influence network impact. WHO grade II (atypical) meningiomas and those that secrete cytokines (VEGF, IL-6) are more likely to generate extensive edema; this corresponds to more severe functional connectivity impairment [1,52]. Thus, a meningioma’s molecular profile (i.e., its propensity for edema via VEGF secretion) is indirectly tied to network disruption [1,52]. Meningiomas seldom have the intratumoral functional heterogeneity of gliomas (since they are extra-axial), but their interface with the cortex can vary; some can invade the cortex and might directly destroy networks, while others remain encapsulated and only compress cortical structures [53]. The former behaves more like a malignancy in terms of network effects, whereas the latter allows the cortex to resume normal function almost immediately after decompression [53].

For brain metastases, histomolecular correlations are less studied, but mechanistic analysis can lead to some interesting insights; for instance, metastases from radiosensitive tumors (like small-cell lung cancer) often present with multiple tiny lesions that are treated with whole-brain radiation, raising the issue of treatment-related network injury (discussed below) [54]. Melanoma metastases, known for hemorrhage, might acutely knock out networks via bleeding (a stroke-like effect) [55]; conversely, some gene-expression subtypes of metastases might be more neurotropic, potentially interacting with neurons through synapse-like connections between tumor cells and neurons [56]. An example is breast cancer or lung cancer cells expressing receptors that respond to neurotransmitters; if present, such tumor-neuron interactions could modulate local network excitability [56,57]. While largely hypothetical at this stage, this represents the frontier of merging molecular oncology with systems neuroscience and could aid in understanding if certain oncogenes influence tumors to integrate into or disrupt neural circuits [57].

In summation, histomolecular features provide a layer of context and complexity for the functional heterogeneity observed in brain tumors. An IDH-wildtype, MGMT-unmethylated glioblastoma causes a rapid, widespread collapse of network function, resulting in low functional connectivity, high structural damage, minimal time for compensatory reorganization, and the ultimate global neurological decline. In contrast, an IDH-mutant, 1p/19q co-deleted oligodendroglioma (slow-growing) might cause networks to rewire and even hyper-connect in some ways, leading to episodic symptoms (seizures) but relatively preserved cognition, and with a potential for near-normal network function after tumor control [58]. Recognizing these patterns can help clinicians anticipate patient needs, such as the indication for aggressive therapy and early cognitive rehabilitation, or the use of anti-epileptic drugs and careful surveillance of cognitive changes over long periods of time after surgical resection.

## 5. Clinical Implications for Surgical Planning and Outcomes

The insights from connectomic and neuroimaging studies of brain tumors carry significant clinical implications. In neurosurgical planning, the goal has evolved from solely maximizing tumor removal to maximizing tumor removal while preserving functional networks, a concept often termed connectome-aware surgery [59,60,61]. Traditional surgical planning relies on identifying “eloquent cortex” (critical areas for language, motor, somatosensory, etc.) via task-based functional mapping, and avoiding those areas [59]. Now, with rs-fMRI, one can map the patient’s networks (like sensorimotor or language networks) preoperatively, which can be especially valuable if the patient cannot cooperate with task-based fMRI [60,61]. A synergy between task-based fMRI and rs-fMRI can aid in the mapping of not only isolated hotspots of function, but also the extent of entire networks and how they might be displaced or reorganized by the tumor [60,61]. For example, preoperative rs-fMRI might reveal that a glioma in the left frontal lobe has caused the language network to shift into the adjacent cortex or to homologous right-hemisphere regions. Briganti et al. studied 39 right-handed patients with left-hemisphere gliomas and 13 healthy controls. global functional connectivity of the language network was significantly reduced in patients compared to controls, particularly in the left temporoparietal junction (TPJ) and in interhemispheric TPJ connectivity (*p* < 0.05) [9]. Knowing this, the surgeon can plan the trajectory and resection boundaries to preserve these new critical areas. A study validating rs-fMRI network mapping in gliomas showed good concordance with intraoperative cortical stimulation mapping, suggesting it can indeed guide safe resection limits [62].

Diffusion tractography, on the other hand, is routinely used to map major white matter tracts around brain tumors (e.g., corticospinal tract, arcuate fasciculus, among others); diffusion imaging has the benefit of analyzing whole-brain structural networks and can aid in the identification of tract disruption or displacement [63]. While not yet standard, some centers construct patient-specific structural connectomes and simulate resection to identify which edges (connections) could be lost. This kind of analysis can highlight, for instance, that resecting an insular glioma will likely sever frontal–parietal connectors and thus risk executive function, even if motor and speech pathways are preserved [43]. By integrating this knowledge, surgical strategy can be adjusted; perhaps a subtotal resection is chosen if a particular hub is too crucial, or extra care is taken during resection when peeling the tumor off a high-connectivity node to minimize collateral damage [63].

Network analysis also has prognostic and predictive value for patient outcomes. Several studies have shown that preoperative connectivity measures correlate with post-operative functional status and even survival [21]. In high-grade gliomas, one group used machine learning rs-fMRI before surgery to predict which patients would regain baseline functional status or present a functional decline after surgery. They found that features of the connectivity matrix, combined with clinical variables, could classify outcomes with promising accuracy [64]. In another analysis, patients with more intact global connectivity before surgery tended to have better overall survival, potentially because their brains could better tolerate aggressive resection or subsequent therapies [65]. This raises the possibility of a “connectomic score” that could inform treatment decisions; for example, if a patient’s brain networks are already highly disrupted by a tumor, one might opt for a more conservative or aggressive surgical approach or prioritize therapies that will not further harm cognition [65].

Another important and often overlooked clinical outcome in brain neoplasms is neurocognitive outcomes several months or years after surgery; these become increasingly important as patients are living longer with tumors. The connectomic perspective reinforces the use of baseline and follow-up cognitive assessments, since network disruption often underlies cognitive deficits even in the absence of focal neurological signs [34]. Interventions such as cognitive rehabilitation can be directed by network findings; for instance, if rs-fMRI shows weakened frontoparietal network connectivity, therapy can focus on tasks recruiting that network to strengthen it (capitalizing on activity-dependent neuroplasticity) [66]. Moreover, the concept of neural reserve and compensation emerges; patients with richer premorbid connectivity or bilateral network representation may cope better with tumor-induced damage [67]. This suggests that encouraging lifestyle factors that promote brain network resilience (like physical exercise and cognitive stimulation) could be beneficial for patients with known brain tumors, akin to building cognitive reserve in neurodegenerative diseases [67].

Certainly, neuropsychological assessments not only demonstrate the cognitive alterations related to a tumor but often help in evaluating the quality of life and the varied cognitive disruptions a patient might experience during their disease or after treatment, especially if linked to surgical procedures [68]. Moreover, neurocognitive function has been proven to be a predictor of patient survival [69]. The connectome perspective, while developed in more recent years, has shown promising results. As shown in Friedrich et al. (2023), cognitive performance is related to structural connectivity across multiple networks. In their study, the authors suggest that, although glioma patients might present similar altered hubs to other conditions like dementia or stroke, the lesions caused by tumors are more heterogenous as they involve damage related to the tumor itself, surgical procedures, metastases, and/or treatment induced damage to white matter which might critically compromise cognitive performance long after [70].

Although the cognitive alterations related to white matter damage have been widely studied for single or isolated functions of neural networks [71], the study of associated hubs and networks shows promising results when treating gliomas, due to the same heterogeneity described above. Certainly, the connectome perspective might be a better approach in gliomas given that association tracts not only link together ipsilateral cortical regions giving rise to specific cognitive functions (e.g., language), but carry this information to other areas related to different cognitive abilities, such as memory, attention, or visuospatial functioning [72]. Therefore, the cognitive evaluation of gliomas cannot be limited to the size or area of the tumor, but to the whole network altered by it. In addition, it has been shown that the location of the tumor only partially explains post-operative cognitive performance [73], and it is pre-operative neuropsychological testing the relevant outperformer, which can even act as a predictor of tumor location and post-surgical cognitive function preservation [74].

From a surgical technique perspective, awake brain surgery with mapping remains the gold standard for protecting critical functions during resection. Connectomics complements this by providing a broader context; while the surgeon tests specific functions intraoperatively, the preoperative connectome can warn of potential deficits in domains that are not easily testable in the OR (attention, social cognition, etc.) by indicating that those networks are at risk [75]. In the future, one could envision real-time intraoperative mapping of functional networks using intraoperative resting-state recordings or even stimulation paradigms that probe network integrity rather than just focal regions. In Table 2, we provide a short summary of the relevant clinical implications that could result from integrating a connectomic and neuroimaging approach to tumor resection.

## 6. Future Directions: Imaging-Guided Therapy and Network-Aware Strategies

**Figure 3:**
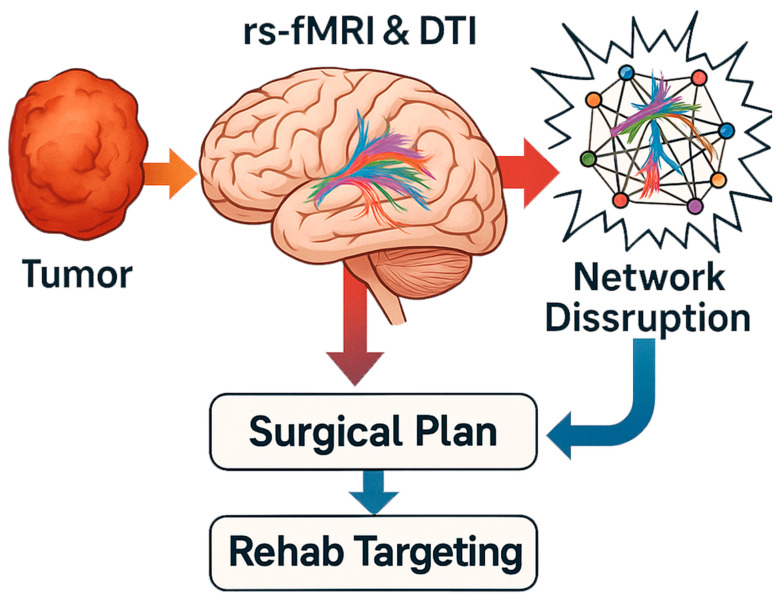
Connectomic applications in neurosurgical planning and prognosis.

As the field progresses, several exciting future directions are on the horizon, aiming to translate the connectomic understanding of brain tumors into better therapies and reduce the surgical footprint on the brain. Figure 3 provides a plausible application map of connectomics and multimodal brain imaging in the surgical management and rehabilitation of brain tumors.

### 6.1. Network-Aware Neurosurgical Navigation

Building on current tractography, future operating rooms may incorporate real-time network visualization. There is ongoing research into augmented reality displays that could project the patient’s functional networks onto the surgical microscope or neuronavigation screen. This would allow the surgeon to see, for example, the margins of the patient’s default mode network or dorsal attention network relative to the tumor in real time and adjust maneuvers accordingly [76]. Standardizing connectome-based surgery protocols and sharing data on functional outcomes will be crucial for validating this approach. Case reports of connectome-guided resection (for instance, in insular gliomas) have shown it is feasible to spare network hubs that might have been routinely sacrificed, thereby preserving nuanced cognitive functions like multitasking or social cognition that traditional mapping might overlook [77].

To move beyond theory, we propose practical pathways for applying emerging connectomic concepts in neuro-oncology. First, for network-aware surgery, preoperative acquisition of rs-fMRI and DTI data can be processed to reconstruct patient-specific functional and structural connectomes. These maps allow identification of reorganized networks, displaced hubs, or contralateral compensatory circuits not apparent on anatomical MRI. Overlaying these networks in surgical navigation systems (or exporting them to neuronavigation platforms) can support surgical planning beyond eloquent cortex avoidance. Second, a connectomic attack refers to the disproportionate functional collapse that occurs when central network hubs are resected. Using graph-theoretical analysis, hubs can be identified (e.g., nodes with high betweenness or eigenvector centrality), and preoperative simulation of resection can estimate the global efficiency drop, aiding risk-benefit assessments of aggressive resections. Finally, functional heterogeneity, defined as the variable impact a tumor exerts on network structure and function, can be modeled by integrating tumor grade, IDH status, connectivity patterns, and lesion-hub proximity. Patients with high functional heterogeneity (e.g., severe structural disconnection but preserved functional coupling) may require tailored rehabilitation or more conservative resection strategies.

### 6.2. Imaging-Guided Radiotherapy

Advances in radiation planning now allow hippocampal-sparing whole-brain radiotherapy (HA-WBRT) for patients with multiple metastases. This technique was developed after trials showed that avoiding radiation dose to the hippocampi during WBRT preserves memory and cognitive function [78]. In the NRG Oncology CC001 phase III trial, patients receiving HA-WBRT plus neuroprotective drugs had significantly less cognitive decline than those with standard WBRT [79]. This is a prime example of a network-aware therapy, as the hippocampus is a key memory hub in the limbic network, and sparing it mitigates damage to the memory circuit [79]. In the future, similar approaches might spare other critical network nodes during radiation; for example, avoiding the cingulate cortex to preserve default mode network function, if feasible, or using proton therapy to reduce scatter to frontal lobe networks [80]. Furthermore, functional MRI could be used to map regions of active cortex that should receive a lower radiation dose, analogous to how we currently spare the motor cortex identified by fMRI.

### 6.3. Restorative Neuromodulation

With better identification of which networks are impaired by a tumor, we can consider therapies to boost network function. Non-invasive brain stimulation techniques like transcranial magnetic stimulation (TMS) or transcranial direct current stimulation (tDCS) could be employed post-surgery to encourage plasticity [81]. For example, if a left frontal tumor resection leaves the patient with weak connectivity in the executive network, intermittent theta-bursts TMS to right frontal homologues might enhance contralesional compensation and improve executive function over time [82]. Similarly, neurofeedback paradigms using fMRI or EEG could allow patients to practice modulating activity in specific networks (e.g., upregulating a sluggish attention network) [83]. These interventions are highly experimental in the tumor setting, but the principle of network-based cognitive rehabilitation is promising.

### 6.4. Connectome-Informed Drug Therapy

An emerging field dubbed “cancer neuroscience” seeks to target the reciprocal interactions between tumors and the nervous system. Some preclinical studies have found that silencing neuronal activity (using chemogenetics or drugs) can slow the growth of infiltrative gliomas, implicating active networks in tumor progression [84]. It is conceivable that in the future, patients could receive adjunct therapies that modulate network activity; for instance, antiseizure medications not just to control seizures but to reduce the hyperexcitability that might be fueling tumor growth [85]. On the flip side, drugs that enhance cognitive network function (like stimulants or cognitive enhancers like ketamine) might be trialed in brain tumor survivors to combat network sluggishness [86]. The challenge will be disentangling direct tumor effects from treatment effects on networks; advanced neuroimaging during clinical trials can help by providing biomarkers of network health.

### 6.5. Personalized Connectome Atlases

Initiatives are underway to collect connectomic data from large cohorts of brain tumor patients (e.g., pre- and post-treatment imaging along with outcomes). This will enable the creation of predictive models; for example, a machine-learning model that takes a patient’s connectome and tumor features as input and outputs the optimal surgical plan or likelihood of recovery in various domains [87]. As data accumulates, such models could become sophisticated “decision support” tools in tumor boards, recommending, say, a subtotal resection if the connectome analysis predicts devastating network loss with total resection, or suggesting additional therapies if a patient’s network shows high risk for a specific cognitive decline [88].

### 6.6. Integration with Molecular Therapies

The connectomic perspective might also influence how we use emerging molecular therapies like targeted inhibitors or immunotherapy. If a certain treatment causes neural side effects (e.g., some immune therapies can induce encephalopathy), monitoring networks via rs-fMRI could catch early signs of toxicity. Conversely, if a drug effectively reduces tumor size, we might see network improvements on fMRI before clinical symptoms change, giving an early indicator of drug benefit [5,89].

In conclusion, future neuro-oncology will likely be a synthesis of molecular precision and functional preservation. Just as tumor genetics guides personalized drug choices, we believe that tumor connectomics will guide personalized brain-restoration strategies. The goal is to extend patient survival and quality of life by treating not only the tumor but also protecting and rehabilitating the brain’s networks. Through interdisciplinary collaboration among neuroimaging scientists, neurosurgeons, neuro-oncologists, radiologists, and rehabilitation experts, these network-aware approaches will continue to develop. The hope is that in the coming years, an ideal brain tumor treatment plan will read something like maximal safe resection assisted by connectomic mapping, tailored radiation sparing key networks, focused medical therapy targeting tumor cells and modulating neural activity, and post-treatment neurorehabilitation aimed at network recovery with an emphasis on quality of life individualized for each patient. This comprehensive approach, rooted in understanding functional heterogeneity, represents the next frontier in conquering the challenges posed by intracranial tumors.

### 6.7. Technical Limitations and Emerging Imaging Solutions

Despite the growing clinical utility of connectomic imaging, several technical limitations remain. Resting-state fMRI, while non-invasive and widely used, is sensitive to physiological noise and motion artifacts, which can compromise signal reliability, particularly in neurosurgical patients who may have altered consciousness or compliance [90]. Similarly, diffusion tensor imaging (DTI), the most common method for mapping structural connectivity, struggles to resolve complex fiber crossings and may misrepresent tract trajectories, especially in regions with high fiber density or tumor-induced distortion [91]. Certainly, technical and physiological confounds can substantially influence connectivity metrics. Head motion remains a critical source of artifact in rs-fMRI, especially in patients with cognitive or motor impairments [90]. Anesthesia during imaging, frequently used in non-cooperative patients, alters neural oscillatory states and can distort resting-state networks, raising interpretive challenges [90]. Additionally, tumor-induced neurovascular uncoupling, particularly in high-grade gliomas, can cause a disconnect between neural activity and the BOLD signal, undermining the reliability of fMRI-based connectivity measures. This phenomenon is especially problematic near tumor margins, where vascular reactivity is compromised, potentially leading to underestimation of preserved functional connections [90]. These limitations can lead to both false positives and false negatives in tractography-based connectome reconstructions. However, recent advances offer promising solutions. Ultra-high-field MRI (7T and beyond) provides improved spatial and temporal resolution for both rs-fMRI and diffusion imaging, enhancing the detection of fine-grained connectivity patterns [92]; furthermore, simultaneous PET-MR platforms enable the integration of metabolic, molecular, and structural–functional data, potentially yielding more precise network-level insights [93]. Moreover, next-generation tractography algorithms, such as constrained spherical deconvolution and anatomically constrained tractography, have demonstrated improved accuracy in delineating crossing fibers and long-range connections [94]. As these technologies mature, they may significantly enhance the specificity and translational reliability of connectome-based assessments in neuro-oncology.

### 6.8. Current Limitations and Research Gaps

While connectomic approaches are increasingly shaping the neuro-oncological landscape, several critical limitations remain that constrain both clinical application and research synthesis. First, there is substantial variability in study designs, imaging modalities, and analytical frameworks across the literature. Differences in MRI acquisition protocols, preprocessing pipelines, and graph-theoretical metrics complicate direct comparison between studies and hinder the development of standardized guidelines. As a result, findings on network disruptions and their clinical correlates may not always be reproducible across centers or cohorts. Second, many clinical applications of connectomics remain experimental. Techniques such as connectome-guided resection simulation, machine-learning–based outcome prediction, and network-targeted neuromodulation show promise but lack prospective validation in large, controlled trials. These tools currently serve more as decision-support systems than definitive guides in standard neurosurgical workflows.

Third, the scientific understanding of maladaptive plasticity in the context of brain tumors is incomplete and requires further investigation. While many studies focus on compensatory reorganization, emerging evidence suggests that some forms of plasticity may be inefficient or even detrimental; for example, network over-simplification, excessive hub activation, or disinhibition-driven seizure networks, as discussed before. Delineating when plasticity is adaptive versus maladaptive remains an important area for longitudinal and mechanistic research. Moreover, the majority of available data are cross-sectional, limiting our ability to infer causal or dynamic relationships between tumor progression, treatment, and network reorganization. Although some longitudinal studies are emerging, there remains a need for prospective imaging–cognition designs that track patients across the disease and recovery trajectory. Compounding this, cognitive outcomes are influenced by a wide range of confounding factors, including pre-existing comorbidities, education level, mood disorders, and medication effects, which are often insufficiently controlled for in connectomic studies.

Ethical and practical considerations also merit attention. The integration of advanced imaging and connectomic data into clinical workflows raises challenges related to interpretation, patient consent, incidental findings, and health disparities in access to high-resolution imaging or computational resources. The increasing use of machine learning for functional prediction based on connectomic profiles further underscores the need for transparency, reproducibility, and robust ethical oversight. This review has primarily focused on gliomas, meningiomas, and brain metastases, which represent the best-characterized tumor types in connectomic research. Other intracranial neoplasms, such as central nervous system lymphomas, embryonal tumors, and ependymomas, remain underrepresented in the literature, especially in the adult population. Additionally, the pediatric connectome introduces unique considerations due to ongoing neurodevelopment, and integrating pediatric tumor data into this framework is a critical next step. Recognizing these limitations is essential not only for contextualizing current findings but also for guiding the next phase of research, which should prioritize methodological standardization, prospective clinical validation, and exploration of underrepresented tumor types and populations.

Finally, although this review primarily focuses on tumor-induced network alterations, it is important to briefly acknowledge the growing body of research on treatment-related network effects. Interventions such as surgery, chemotherapy, radiotherapy, and even immunotherapy can independently modify brain connectivity, often compounding or interacting with tumor-related changes. For instance, whole-brain radiotherapy is known to disrupt default mode and frontoparietal network connectivity, particularly in older patients or those with preexisting comorbidities [95]. Chemotherapeutic agents like temozolomide may induce white matter changes and alter functional connectivity in long-term glioma survivors [96], while newer immune checkpoint inhibitors have been linked to paraneoplastic encephalopathy with diffuse network dysfunction [97]. These effects are increasingly detectable through longitudinal rs-fMRI and diffusion MRI studies, reinforcing the need for network-aware monitoring throughout the treatment trajectory. As such, incorporating functional connectomics into treatment planning and follow-up may help mitigate adverse effects and tailor rehabilitation strategies more precisely.

## 7. Conclusions

Intracranial tumors fundamentally challenge the brain’s connectome, each in its own way. Diffuse gliomas insinuate themselves into neural circuits, eroding the structural scaffolding of networks and dampening functional connectivity, yet sometimes using those same networks to support their growth or to enable brain plasticity around them. Meningiomas and metastases, though more focal, send ripples through brain networks via edema, pressure, and remote effects, underscoring that even a localized lesions have system-wide ramifications. By leveraging advanced neuroimaging and connectomic analysis, we can now visualize and quantify these effects, from disrupted resting-state networks and altered graph metrics to signs of compensatory reorganization. Appreciating this functional heterogeneity is not just an academic exercise, but a clinical imperative. It allows us to anticipate which patients may experience cognitive decline, to plan surgeries that balance resection with network preservation, and to design adjunct therapies that harness brain plasticity.

The literature reviewed herein spans classic studies of tumor-related diaschisis to cutting-edge connectome research, converging on a key message: the brain-tumor interface is as much functional as it is structural. A tumor’s impact cannot be fully understood or managed without considering the networks it disrupts and the brain’s attempt to compensate. Future treatments that explicitly integrate connectomic insights, such as network-sparing surgical trajectories, hippocampal-avoidance radiation, or post-operative network rehabilitation protocols, hold great promise for improving outcomes. As we continue to decode the connectome in neuro-oncology, we move closer to an era where we not only fight the tumor but also heal the brain. Certainly, by keeping the whole-brain perspective in focus, clinicians and researchers can ensure that advances in molecular medicine are complemented by equal advances in preserving the mind. In doing so, we honor the principle that saving the brain means more than just removing the tumor but also safeguarding the dynamic web of connections that underlie an individual’s cognitive life.

## Figures and Tables

**Table 1:** Overview of functional connectome disruption, reorganization, and clinical implications by tumor type.

Key Area	Tumor Type	Mechanisms of Network Disruption	Reorganization Patterns	Histomolecular Influences	Clinical Implications
Tumor-Specific Patterns	Gliomas	Infiltration along white matter tracts; long-range disconnection; hub disruption	Partial functional preservation; structural–functional decoupling; contralesional compensation	IDH-wildtype: severe disconnection; IDH-mutant: partial preservation	Functional connectivity may inform prognosis and surgical planning
	Meningiomas	Mass effect; edema-induced disconnection; cortical compression	Partial post-resection normalization; gradual cognitive recovery	VEGF-driven edema; pial blood supply affects FC loss	Even benign cases with edema can show global FC loss; cognitive testing recommended
	Metastases	Focal disruption; diaschisis; network fragmentation with multiple lesions	Limited reorganization; interhemispheric imbalance	Subtype-specific neurotropism; molecular profiles under study	Multiple lesions increase cognitive burden; planning around hubs essential
Post-surgical Plasticity	All	Surgery disrupts hubs, tracts, and connectivity nodes	Rewiring via contralesional recruitment, local reactivation, and modular reformation	Post-IDH surgery recovery better; FC rebound linked to histology	Recovery depends on pre-op connectivity; tractography aids surgical strategy
Histomolecular	Gliomas	Varies by IDH, 1p/19q, MGMT; influences severity of disconnection	IDH-mutant allows better network preservation; plasticity potential higher	Histology informs prognosis and network vulnerability	Histomolecular profile predicts recovery and guides treatment intensity
	Meningiomas	Cortical invasion rare; edema modulated by VEGF, IL-6	Minimal plasticity unless cortical invasion	VEGF, IL-6 secretion linked to edema and FC disruption	Invasive meningiomas resemble gliomas in FC terms; more aggressive care warranted
	Metastases	Tumor-neuron synapses; hemorrhage (e.g., melanoma)	Sparse data; limited evidence of large-scale reorganization	Tumor origin (e.g., breast, lung) may affect neuron interaction	Profile-specific predictions of network disruption emerging

**Table 2:** Major clinical implications of connectomic and neuroimaging for surgical planning and outcomes.

Main Takeaways
1. **Redefinition of Surgical Goal:** Focus on maximal tumor resection while preserving functional brain networks during surgery (functional/connectome-aware neurosurgery).
2. **Preoperative Mapping:** rs-fMRI allows mapping of whole functional networks, even in those unable to cooperate with task-based imaging or examination.
3. **Diffusion Tractography and Structural Connectomes:** Identification of white matter tracts and simulation of resections help guide surgical planning by revealing critical networks that should be preserved.
4. **Prognostic Value of Connectivity:** Preoperative brain connectivity correlates with postoperative functional outcomes, survival, and rehabilitation, making it a valuable tool for guiding treatment decisions.
5. **Awake Surgery and Connectomics Integration:** Awake mapping safeguards key functions, while preoperative connectome data highlights risks to broader, non-testable networks.

## Data Availability

Not applicable.

## Abbreviations

The following abbreviations are used in this manuscript:

2-HG	2-Hydroxyglutarate
ANCOVA	Analysis of Covariance
AUC	Area Under the Curve
BOLD	Blood-Oxygen-Level Dependent
cSMN	Contralesional Sensorimotor Network
DMN	Default Mode Network
DTI	Diffusion Tensor Imaging
EEG	Electroencephalography
FC	Functional Connectivity
GBM	Glioblastoma
HA-WBRT	Hippocampal-Avoidance Whole-Brain Radiotherapy
IDH	Isocitrate Dehydrogenase
IL-6	Interleukin 6
MEG	Magnetoencephalography
MGMT	O6-Methylguanine-DNA Methyltransferase
MoCA	Montreal Cognitive Assessment
PET-MR	Positron Emission Tomography—Magnetic Resonance
PTBE	Peritumoral Brain Edema
rs-fMRI	Resting-State Functional Magnetic Resonance Imaging
RSNs	Resting-State Networks
SC	Structural Connectivity
tDCS	Transcranial Direct Current Stimulation
TMS	Transcranial Magnetic Stimulation
TPJ	Temporoparietal Junction
VEGF	Vascular Endothelial Growth Factor
WBRT	Whole-Brain Radiotherapy
WHO	World Health Organization
